# Are Serotonin Alterations the Link between Thrombocytopenia and Poor Immune Status among HIV Infected Individuals?

**DOI:** 10.4172/2155-6113.1000277

**Published:** 2014-02-10

**Authors:** María José Míguez-Burbano, Allan Rodriguez, Mayra Vargas, Gabriella Tantalean, Ranjini Valiathan, Wenyaw Chan

**Affiliations:** 1 School of Integrated Science and Humanity, Florida International University, Miami, FL, USA; 2 Department of Medicine, University of Miami School of Medicine, Miami, FL, USA; 3 Department of Psychiatry & Behavioral Sciences, University of Miami School of Medicine, Miami, FL, USA; 4 Division of Biostatistics, School of Public Health, University of Texas-Health Science Center at Houston, USA

**Keywords:** Thrombocytopenia, BDNF, Serotonin, Alcohol, HIV/AIDS

## Abstract

**Objective:**

Thrombocytopenia (TCP<150 × 10^3^ cells/mm^3^) has emerged as a relevant factor in the clinical course of HIV. However, the mechanisms mediating such observations have not been well characterized, limiting the possibility of creating targeted interventions. Notably, platelets are the storage and transporter system for serotonin and Brain derived neurotrophic factor (BDNF), which recent laboratory studies associated with viral replication and lymphocyte survival. Thus, we posit that (1) TCP will be associated with reduced levels of BDNF and serotonin (2) That these alterations will lead to poor viro-immune responses to antiretroviral therapy.

**Methods:**

To achieve this goal, a total of 400 people living with HIV were consecutively enrolled to characterize the frequency of thrombocytopenia in hazardous and non-hazardous alcohol user populations in the HAART era. Then, participants underwent immune and laboratory assessments, to determine if TCP was associated with alterations in serotonin (5-HT) and brain derived neurotrophic factor (BDNF).

**Results:**

The prevalence of thrombocytopenia in this antiretroviral treated cohort was 14%. Rates were significantly higher in the heavy alcohol users, HAU versus the non HAU group (Heavy: 25% versus HAU: 15% versusnon-HAU: 10%). Multivariate model analyses indicated that having TCP, low BDNF levels (<5000 pg/ml), and number of drinks per day were predictors of serotonin levels. PLWH with TCP had about 2-fold lower PPP-BDNFlevels (5037.4 ± 381 vs. 9137.5 ± 7062 pg/ml *p*=0.0001). Other significant predictors of BDNF levels at the last visit included receiving selective serotonin reuptake inhibitors and PPP serotonin levels. Multivariate analyses also confirmed that altered serotonin levels were associated withhigh viral loadsboth low CD4 cell counts.

**Conclusions:**

Thrombocytopenia is a relatively frequent complication of HIV, andis particularly prevalent among hazardous alcohol users (HAU). These findings suggest that TCP is associated with altered levels of BDNF and serotonin, suggesting that they may be the bridge linking TCP and poor viro-immune responses observed in this group. These results could have important clinical and therapeutic implications.

## Introduction

Thrombocytopenia (TCP: a platelet count of less than 150,000 per microliter), is a common hematological complication which affects a sizable proportion of people living with HIV (PLWH) [1-6]. Although TCP causes are multifactorial, given the direct effect of HIV in platelet destruction, it was expected that this problem would no-longer exist with the introduction of antiretroviral therapy. However, thrombocytopenia is still present in approximately 15% of the cases [1-6]. Our prior study indicates that alcohol negatively impacts platelets, and may be an important contributing factor to TCP persistence [6,7].

Although frequently overseen, TCP hasbeen associated with increased morbidity and mortality [1-4]. Nevertheless, the mechanism mediating this association is uncertain, highlighting the need for further research. In this regards, it needs to be emphasized that the role of platelets has expandedfrom hemostasis to inflammation, and host defense [8,9]. In fact, confluent pieces of evidence indicate that platelets share structural and functional similarities with granulocytes, known to participate in antimicrobial host defense. Platelets have the ability to internalize the HIV, suggesting a potential role of platelets in the immune response [8,9]. Notably, platelets also contain a number of broad spectrum antimicrobial peptides, RANTES and MIP-1 that can enact as HIV-suppressive factors [8,9].

**Figure 1:**
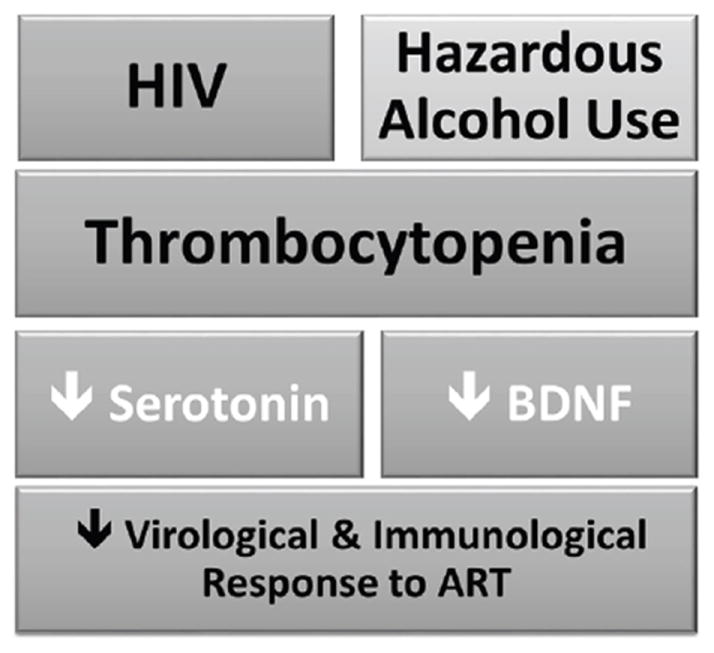
Proposed Model.

Circulating platelets may enact as a transportation system for serotonin (5-hydroxytryptamine, 5-HT) [10-14]. Serotonin is aneuromodulator, stored within the dense granules of the platelets, that only becomes physiologically active upon platelet degranulation [10-11,14]. Platelets are also a source of brain derived neurotrophic factor (BDNF), for which an auto/paracrine feedback loop regulation with serotonin exist. Notably, recent studies have uncovered important roles for serotonin in the periphery, including liver regeneration, and support to the immune response [14]. Laboratory experiments have demonstrated that serotonin is needed for T cell proliferation [15]. Monocytes/macrophages which are involved in the control of HIV also express serotonin receptors. Relevant for our proposal, studies also suggest that 5-HT can inhibit the replication of HIV-1 in primary culture of human macrophages through its action on 5-HT1A receptors [16]. Therefore, it is of great interest to assess among PLWH the impact of TCP and serotonin in the periphery. As depicted in Figure 1, we postulate *a priori* that thrombocytopenia will be accompanied with BDNF and serotonin disorders that may impact the immune response as well viral control.

## Methods

### Sampling

The Platelets Mediating Alcohol and HIV Damage Study (PADS) is a large, single-site multi-ethnic cohort, consisting of 400 PLWH, who are at least 18 years old and under regular care at Miami’s primary open-access public health system. Participants were recruited via flyers, personal contact at the clinics, or called our office to schedule an appointment. Our choice of PLWH in an open-access public health system with standard treatment protocols was purposefully designed to minimize social, medical, and treatment inequalities. To reduce the confounding effects of illicit drug use, the DSM-IV-TR questionnaire was applied, and those who were dependent on drugs or injecting illicit psychoactive substances were excluded.

Non-ambulatory patients, and those presenting with major medical co-morbidities, such as CNS opportunistic infection, head injury, tumors, major psychiatricdisease, developmental disorders, severe malnutrition, chronic renal failure, intestinal pathology, thyroid problems, cardiovascular or immune-based disease, (i.e., malignancies, autoimmune diseases, or arthritis) were excluded. In addition, based on medical records, participants who had cirrhosisor active viral hepatitis were not eligible. Otherwise, the subject was enrolled.

PADS were approved by the central governing Institutional Review Boards at Florida International University and University of Miami. The study was conducted according to the principles expressed in the Declaration of Helsinki. Those participants who provided written informed consent and a signed medical release form were consecutively enrolled, and followed over a period of six months.

### Alcohol use

At each visit, PLWH reported alcohol intake in the past six months, using two standardized and validated brief screening questionnaires: the Alcohol Use Disorders Identification Test (AUDIT), and the Alcohol Dependence Scale (ADS) [17-19]. Alcohol consumption scores were computed by averaging cross products of quantity and frequency of beer/wine and hard liquor reported on the AUDIT and ADS responses. Then, based on the National Institute of Alcohol Abuse and Alcoholism guidelines criteria, men who reported >14 drinks/week or >4 drinks in one day, and women>7 drinks/week or >3 drinks in one day were classified as HAU, while those who reported fewer drinks were categorized as non-HAU [19].

### Platelet counts, brain derived neurotrophic factor, and serotonin

Blood was drawn in fasting subjects in order to best evaluate immunological, andhematological parameters. Cell blood counts were obtained using the cell-Dyn 4000, a multi-parameter automatedhematology analyzer system, recommended for specimens with low or high plateletconcentrations. Thrombocytopenia (absolute thrombocytopenia) was defined as platelet counts below 150 × 10^3^ cells/mm^3^. Subjects with PLT counts above this threshold served as the reference group.

Platelet-poor plasma (PPP) was obtained, as it is commonly used to measure platelet associated factors. To obtain platelet-poor plasma, blood samples collected in EDTA-coated tubes (plasma) (BD Diagnostic Systems, NJ, USA) were stored in ice. Plasma was separated by centrifugation at 40°C for 15 min at 1,500 × g. This plasma was again re-centrifuged at 10,000 × g and aliquots of PPP were stored in polypropylene tubes at -80°C until assayed.

Quantification of serotonin (5-HT) was achieved using a commercially available enzyme-linked immune sorbent assay (ELISA), and procedures were followed asper the manufacturer’s instructions (GenWay Biotech, San Diego, CA). PPP BDNF levels were measuredusing an ELISA kit (R&D System), according to the manufacturer’s instructions. Briefly, 50 μl of standards and 20 fold diluted samples were pipetted into wells of 96-well immune plates. An enzyme-linked monoclonal antibody specific for BDNF was added to the wells. The maximum detectable dose of BDNF is typically 4,000 pg/mL, but samples were further diluted because the majority of study participants had values above 4,000 pg/ml.

### Viral load and CD4 cell outcomes

Flow cytometry was used to quantify the percentage and absolute numbers of T lymphocyte sub populationsCD3+/CD4+ and CD3+/CD8. In addition, HIV viral burden was quantified using the Amplicor HIV monitor test (Roche Diagnostic System). Virological success was defined as achieving undetectable viral loads.

### Covariates

Upon entry into the study, and using standardized questionnaires, sociodemographic data, medical history, HIV- related variables including years since HIV diagnosis, AIDS diagnosis, antiretroviral regimen and duration, adherence, treatment for mood disorders, and liver-related testswere obtained.

### Statistical analyses

A sample size calculation estimated that current sample size would provide 80% power to detect differences between platelet groups (alpha of 0.05). The data were analyzed using SAS version 8 and SPSS version 11, and *p* values<0.05 were considered to be statistically significant. All variables were examined for skewness, and when necessary were log-transformed (to base 10; such as viral load). Following descriptive statistical analyses, baseline demographic, clinical, BDNF and serotonin differences between the two platelet groups were compared using Student’s t test and one-way analysis of variance. Correlations among the main variables of interest wereexamined with Pearson’s coefficients.

Univariate, bivariate and multivariate analyses were used to calculate odds ratios (OR) and 95% confidenceintervals (CI). Logistic regression analyses were used to evaluate the effects ofalcohol (hazardous vs. non-hazardous), and thrombocytopenia (thrombocytopenia vs. normal platelet counts) on BDNF and serotonin levels, while controlling for age, gender, race, and receiving therapy for neuropsychological disorders. Potential predictors of virological and immune responses (i.e., gender, race/ethnicity, CDC status, druguse, and body mass index) were selected on the basis of the HIV medical literature, andwere added to the model. Non-significant variables (*p*≥0.05) were removed, beginningwith the least significant variable, until the final full model was achieved.

## Results

### Study population characteristics

Most (80%) of the individuals in the study had normal platelets, hemoglobin, andhematocrit measurements, and no significant differences in mean hemoglobin, hematocrit, or proportion of anemia were observed between patients with thrombocytopenia (13.2 ± 2.0) and those with normal platelet counts (13.0 ± 1.9 g/dl).

As depicted in Table 1, thrombocytopenic patients were more likely to be males(22% vs. 7%, *p*=0.0001). They also tended to have higher AST (*p*=0.06), but did not differ in ALT values. HIV-associated thrombocytopenia occurred in patients from all major risk groups, including those exposed via homosexual or heterosexual contact, injection drug use, transfusions and those with multiple risks. Yet, additional analyses indicated that thrombocytopenic patients significantly differed in their patterns of alcohol consumption, when compared to patients with normal platelet counts. Subjects with thrombocytopenia consumed in average more drinks per week. Additional analyses indicated that they were more likely to be hazardous alcohol users (*Odds Ratio*: OR=2.4, 95% CI: 1.2-7.2, *p*=0.05). They also had slightly more years drinking (2 years), though differences did not reach statistical significance. Notably, groups did not differ in age or socioeconomic status. Groups were similar in the number of years living with HIV, type of treatment received and the proportion of subjects being adherent to the prescribed regimens.

### Thrombocytopenia and BDNF

To evaluate whether there was a relationship between platelet counts and serotonin levels we started by running correlations. Analyses revealed a significant correlation between platelet counts and 5HT levels (r=0.14, *p*=0.01). Among PLWH, a main effect was observed in the platelet group, with subjects with normal platelet counts having significantly higher serotonin levels were significantly reduced in PLWH with TCP, as compared to those with low platelet counts (89.4 ± 5.4 vs.45.9 ± 7.6 ng/mL, *p*=0.001). Group differences were substantial; mean serotonin levels of the TCP groupwere 52% lower than the control group. Serotonin levels tended to differby gender among PLWH, with women exhibiting the highest levels (93.6 ± 7.8 vs. 76.9 ± 5.5 ng/ml, *p*=0.07).

### Serotonin and treatments

Given that antidepressant treatment mechanisms of action have been linked withregulations of serotonin levels, we investigatedif these treatments may overcome the observed changes. Levels of PPP serotonin were lower in subjects receiving selective serotonin reuptakeinhibitors (SSRIs), (62.4 ± 9.7 vs. 88.6 ± 5.0 ng/mL*p*=0.02). Viral loads were notably lower in patients in the SSRI group when compared to the non-SSRI group (2.5 ± 1.2 vs. 2.8 ± 1.3 viral log, *p*=0.04).

Based on prior studies indicating that HIV directly can affect serotonin, we examined the impact of antiretroviral treatment on serotonin levels [20]. In accord with prior studies, differences in PPP serotonin levels were found between patients who were receiving antiretrovirals and achieved viral suppression and those who did not (undetectable: 98.0 ± 6.6 vs. 68.9 ± 6 ng/mL, *p*=0.001).

### Longitudinal analyses

When a longitudinal analysis was performed, platelet status was a significant predictor of serotoninandBDNF. Serotonin did not significantly change during the length of the study in TCP subjects, yet among those with normal platelet counts a decrease in both PPP serotonin (-31 ± 7.7 ng/mL, *p*=0.0001) and BNDF (-1423 ± 754, pg/ml, *p*=0.06) was observed during the 6 month follow-up.

In a multiple regression model (see Table 2), with serotonin levels at the last visit as the dependent variable, there were no significant contributions from gender (*p*=0.61), race (*p*=0.38), BMI, or receiving antidepressants (*p*=0.1). Yet, platelet count below 150 ×10^3^ cells/mm^3^ (*p*=0.05), BDNF below 5000 pg/ml (*p*=0.0001), and number of drinks per day were significant predictors (*p*=0.008).

### Clinical relevance

To evaluate whether there was a correlation between platelet counts and viro-immune status, we first compared the mean CD4 cell counts and viral loads of patients with and without TCP. Subjects with TCP had significantly lower CD4 counts both at baseline (307 ± 237 vs.450 ± 284 cell counts, *p*=0.001); and at follow-up(275 ± 230 vs.426 ± 275 cell counts, *p*=0.005). Individuals with TCP also had higher viral loads at baseline (3.3 ± 1.5 vs. 2.6 ± 1.3 viral load log,*p*=0.02). Viral loads at the follow-up assessmentwere also higher (3.4 ± 1.6 vs. 2.7 ± 1.3 viral load log, *p*=0.05).

Finally, using Linear Regression analyses, we investigated whether differential CD4 and viral load responses observed in people receiving antiretrovirals may be associated with the status of serotonin and TCP. Multivariate logistic regressions were fitted to predict the probability of having less than 500 CD4 cell counts at the last visit by considering the following as explanatory variables: age, gender, race, route of transmission, time with HIV infection, time receiving antiretroviral therapy, antiretrovirals used at baseline, adherence, viral load, alcohol use, BMI, albumin, and liver function (Tables 3 and 4). The first regression model examined the predictors of CD4 cell counts above 500 at the last visit, and the *R^2^* was 0.29, indicating that the model predicted approximately 29% of the variance in the cell counts (In the multivariate analysis (Table 4), total drinks per week and gender were the only significant predictors of CD4 cell countsat the last visit.

The second model focuses on viral load suppression at the last visit. The *R^2^* of the model was 0.23. Analyses confirmed that TCP, age, baseline CD4 counts, serotonin levels and SSRI are important predictors of successful suppression of HIV.

## Discussion

First, our data demonstrated that having TCP is still common (15-25%) in our urban HIV population in South Florida. TCP occurs indistinctively in those exposed via injection drug use, blood transfusion, or by homosexual or heterosexual contact. Yet it climbs as high as 25% among heavy alcohol users. In accord with our initial hypothesis, analyses revealed positive associations between TCP and abnormal serotonin and BDNF values,indicating that we should redirect our concerns to ensure that PLWH are maintaining a normal platelet count. Regression analysis revealed that alterations in serotonin levels were significantly associated with antiviral therapy outcomes. These results have several public health and clinical implications. The data has provided first time evidence of the mechanism mediating the observed relationship between TCP and poor clinical prognosis. These findings underscore the need for treatment of hematological and mood disorders if antiretroviral therapy is to be fully successful. This studyhas also set the stage for new clinical trials aiming to improve platelet counts, given the limited therapeutic options currently available for PLWH with these hematological disorders. Though we focused on virological and immune responses, given BDNF and 5HT critical roles on cognitive and mood homeostasis [14,15], the need to closely follow-up TCP subjects is clear.

Prevalence estimates of thrombocytopenia (<150000 platelets/l) in the literature varied greatly (10-30%); however, after the introduction of antiretroviral therapy, prevalences have been generally above 10% [1-6]. Nevertheless, in our urban HIV population in South Florida and despite having received therapy, TCP was present in 15% of the sample. In other words, if worldwide there are 35.3 million people living with HIV [21], then between 4-6 million of them could develop TCP. Such excessive rates should receive more attention, given TCP’s confirmed role on clinical morbidity and mortality in PLWH [1-6]. Although many scientists and clinicians are only linking TCP with vascular problems, our analyses confirmed that TCP was strongly associated with uncontrolled HIV replication. Analyses also highlighted the relevance of platelets in achieving high CD4 cell counts and a stronger overall on the immune response. However, much work still needs to be performed to identify precisely what role platelets have in the HIV disease beyond the cardiovascular system.

We found serotonin deficiencies observed amongsubjects with thrombocytopenia. Indeed,in the multivariate model BDNF concentrations, TCP and high viral loads were the only predictors of serotonin levels at the last visit. The concomitant presence of thrombocytopenia and alcohol could be reducing the supply of tryptophan and serotonin production [22]. In addition, BDNF depletions can be compromising the function and growth of serotonergic neurons [23]. Beyond HIV specific risks, many neuropsychological conditions have a serotonergic component, and therefore it could be expected that these subjects will be at risk of developing these types of disorders [15]. Serotonin and platelet growth factors are also needed to promote liver regeneration, a response that will be limited in alcohol users and older subjects [15]. Accordingly, correcting platelet, serotonin and BDNF alterations is of medical importance, particularly as this population ages.

Serotonin’s role in viral control deserves special consideration. We discovered differences in PPP serotonin levels between subjects who achieved viral suppression and those who did not. The decreased levels of serotonin among TCP subjects are of great concern, given recent studies demonstrating *in vitro* that 5-HT is able to decrease HIV infection via its action on 5-HT1A receptors [17,24]. In addition, serotonin decreased the expression of the HIV co-receptor CCR5, and increased MIP-1α [17,24]. Notably, subjects taking SSRIs displayed significantly lower viral load levels, suggesting that SSRIs, by increasing extracellular concentrations of serotonin may suppress HIV infectivity and replication. These findings also warrant intervention studies to determine if other drugs that target serotonin and BDNF maybe useful as supportive therapy.

Nevertheless, our results should be interpreted in the context of the study limitations: First, a short time follow-up that reduced our capacity to perform more detail analyses. Second, the study designdid not allow to establish causality, neither to generalize study findings as we primarily focusedon alcohol. Therefore, additional studies are needed to replicate our findings among subjects with viral hepatitis and with drug abusers. Nonetheless, these results may have important research, clinical, and therapeutic implications. They improve our understanding of the relevance of platelets in the neuro-immune systems. By identifying those HIV-infected individuals at risk for ART failures, such as those with thrombocytopenia, alcohol abuse, and with BDNF and serotonin deficits the study may permit early clinical follow-ups and interventions. Findings may also guide the development of future therapies.

## Figures and Tables

**Table 1:** Sample Characteristics by TCP Group.

Variable	Non-TCP (N=80)	TCP (N=320)	*P value*

**Age**	43 ± 6.7	42.6 ± 7	0.8

**Gender**			
Men	56% (45)	74% (236)	0.01
Women	44% (35)	26% (84)	

**Race/Ethnicity**			0.1
African American	63% (50)	73% (234)	

Black Caribbean	3% (2)	2% (6)	

Hispanic	27% (22)	20% (64)	

White	7% (6%)	5% (16)	

**Education** (years of school)	11.6 ± 2.3	11.3 ± 2.3	0.2

**Years Living with HIV**	14.9 ± 7.5	15.6 ± 8	0.6

**Mode of HIV Transmission**			0.2
Homosexual	59%	51%	
Heterosexual	30%	26%	
Drugs	3%	8%	
Transfusion	4%	8%	
Multiple risks of Exposure	4%	7%	

**Years drinking**	6.6 ± 1.2	8.8 ± 0.6	0.1

**Total number of drinks per week**	15 ± 1.3	19 ± 3.5	0.04

**Albumin** mg/dl	4 ± 0.6	4.2 ± 0.5	0.9

**Liver enzymes**			
AST IU/L	35 ± 20	41 ± 23	0.06

ALT IU/L	33 ± 30	38 ± 27	0.3

**Antiretroviral Therapy**	94%	93%	0.5

**Adherence (ACTG)**	86%	88%	0.5

**Mood disorders treatment**	32%	37%	0.2

Note. Values are means ± SD or percentages with exact n in the parenthesis.

Note. Significant drinking and gender differences were found between groups.

**Table 2:** Multivariate Analyses of Serotonin (cut off 50).

		OR	95% Confidence Interval		

			Lower	Upper	P value

1	(Constant)				

	Non-TCP	0.71	0.308	1.63	0.05

	BDNF	0.142	0.076	0.266	.0001

	Binge Drinking	0.400	0.211	0.761	.0052
	SSRI	0.338	0.400	0.211	0.008

Partition for the Hosmer and Lemeshow Test (*p*=0.26).

TCP: a platelet count of less than 150,000 per microliter. BDNF: Brain Derived Neurotrophic Factor. SSRI: Selective serotonin reuptake inhibitors.

**Table 3:** Multivariate analyses of predictors of undetectable viral loads in subjects receiving antiretroviral therapy.

Parameter	OR	95% Confidence Interval		
		Lower	Upper	Sig.
CD4 Counts <500	0.322	-112.302	-45.654	0.000
Binge Drinking	0.335	1.211	7.96	0.047
No-TCP	8.7	5.50	11.301	0.000
High Serotonin	5.479	2.504	11.991	0.05

Partition for the Hosmer and Leme show Test (*p*=0.26).

**Table 4:** Multivariate Analyses for CD4 counts above 500 at the last visit.

Variables	OR	Significance	95% CI Lower Bound	95% CI Upper Bound
Viral Load undetectable	17.772	0.000	4.964	63.626
Gender	3.80	0.001	2.1	7.39
No Binge Drinkers	1.939	0.026	1.689	4.908

Partition for the Hosmer and Leme show Test (*p*=0.8).
